# A21 OVEREXPRESSION OF *ASCL2* ALTERS DIFFERENTIATION IN ESOPHAGEAL ORGANOIDS

**DOI:** 10.1093/jcag/gwab049.020

**Published:** 2022-02-21

**Authors:** M Hamilton, D Jean, F Boudreau, V Giroux

**Affiliations:** Immunologie et Biologie cellulaire, Universite de Sherbrooke, Sherbrooke, QC, Canada

## Abstract

**Background:**

The first population of stem cells in the esophageal epithelium was recently identified with the marker *Keratin 15* ( *Krt15*). However, little is known about the mechanisms underlying the expansion and the function of these stem cells. It was shown that the transcription factor ASCL2 is upregulated in *Krt15+* cells compared to *Krt15*- cells. Interestingly, ASCL2 is a gene target of the Wnt/β-catenin pathway, which acts as a regulator of proliferation and maintenance of the stemness state. The ultimate goal of my research project is to determine the role of ASCL2 in the maintenance of esophageal stem cells and to identify its binding partners.

**Aims:**

Investigate the role of ASCL2 in esophageal epithelial biology.

**Methods:**

Lentiviral infection approach was used to obtain mouse esophageal organoids overexpressing ASCL2. Organoid culture, immunostaining (such as IF and H&E), qPCR, WB and proliferation assay were used to characterize the effect of ASCL2 overexpression on morphology, differentiation, proliferation, self-renewal and gene expression.

**Results:**

First, ASCL2 overexpression was confirmed by WB. Interestingly, the morphology of organoid overexpressing ASCL2 was severely altered: organoids were smaller and less differentiated. Defect in differentiation was investigated by qPCR and IF using relevant markers such as *p63*, *Krt13*, *Wnt5a* and *NT5E*. Indeed, we observed an increase in basal marker ( *p63*), a decrease in suprabasal markers ( *Krt13, Wnt5a*) and in a stem cell marker ( *NT5E*). We also investigated the role of ASCL2 in self-renewal and observed that organoid formation capacity was reduced in ASCL2-overexpressing organoids. Furthermore, proliferation was also reduced in WST-1 assays. We also observed lower expression of the gene *Top2a*, a recently identified marker of the proliferative basal cell population in the human esophagus. Finally, we observed significant changes in the expression of genes associated with quiescent stem cells (Clu, ZFP36L2 and Anxa1).

**Conclusions:**

ASCL2 overexpression alters differentiation and proliferation in organoids. ASCL2 could play a role in orchestrating cell fate decision in the esophageal epithelium.

**Funding Agencies:**

NSERC, Canada Research Chair

